# Study protocol for a multicenter, multinational prospective randomized controlled trial comparing outcomes in subjects with Gram-negative bacteremia who have blood culture evaluation using *F*ast *A*ntibiotic *S*usceptibility *T*esting vs. standard of care testing: the FAST trial

**DOI:** 10.1186/s13063-025-09228-4

**Published:** 2025-11-12

**Authors:** Ritu Banerjee, Lauren Komarow, Yixuan Li, Qihang Wu, Lucia Sanchez-Gonzalez, Donald Mau, Erin Abbenante, Nyssa Schwager, Andrew Dodd, Maria Souli, Holly S. Geres, Sarah Doernberg, Kerryl Greenwood-Quaintance, Scott R. Evans, Henry F. Chambers, Vance G. Fowler, Robin Patel, Antibacterial Resistance Leadership Group

**Affiliations:** 1 Department of Pediatrics, Vanderbilt University Medical Center, Nashville, TN USA; 2 The Biostatistics Center, George Washington University, Rockville, MD USA; 3 Duke Clinical Research Institute, Duke University Medical Center, Durham, NC USA; 4 Department of Medicine, University of California, San Francisco, CA USA; 5 Department of Laboratory Medicine and Pathology, Mayo Clinic, Rochester, MN USA

**Keywords:** Bloodstream infection, Antibiotic susceptibility testing, Gram-negative, Bacteremia

## Abstract

**Background:**

Novel, rapid blood culture diagnostics can provide faster antibiotic susceptibility results (AST) compared to standard methods but their impact on clinical outcomes is unclear and not assessed in many prospective clinical trials.

**Methods:**

This study is a two-arm, multicenter, multinational, prospective randomized controlled clinical trial conducted in countries with high antibiotic resistance rates including Greece, India, Israel, and Spain. Nine hundred hospitalized subjects who have blood cultures collected as part of routine clinical care with growth of Gram-negative bacilli (GNB) will be randomized 1:1 to blood culture evaluation using standard of care (SOC) AST vs. a rapid phenotypic AST method, VITEK REVEAL™ in addition to SOC AST. Subjects in both study arms will be reviewed by antibiotic stewardship clinicians who will recommend changes to antibiotic therapy, if indicated. The primary outcome is a composite three-category Desirability of Outcome Ranking (DOOR) defined using three ordered levels: alive without deleterious events, alive with at least one deleterious event, and death. Key secondary outcomes include mortality, length of stay, and time to antibiotic escalation or de-escalation within 3 days of randomization. Exploratory outcomes include a five-category DOOR, categorial agreement between VITEK REVEAL™ and SOC testing, clinician compliance with antibiotic stewardship recommendations, and desirability of treatment selection based on antibiotic spectrum, activity, and bloodstream isolate susceptibility profile (DOOR MAT). The primary analysis will be conducted on the modified intention-to-treat population.

**Discussion:**

This trial will evaluate whether use of a rapid phenotypic AST method improves outcomes compared to use of conventional methods for patients with Gram-negative bloodstream infections in clinical settings with high antibiotic resistance rates.

**Trial registration:**

ClinicalTrials.gov ID NCT06174649. Registered on Dec 18 2023.

**Protocol version:**

Number 20-0021, version 5.0, 11-April-2024.

## Administrative information

Note: the numbers in curly brackets in this protocol refer to SPIRIT checklist item numbers. The order of the items has been modified to group similar items (see http://www.equator-network.org/reporting-guidelines/spirit-2013-statement-defining-standard-protocol-items-for-clinical-trials/).

**Table:** 

Title {1}	A multicenter, multinational prospective randomized controlled trial comparing outcomes in subjects with Gram-negative bacteremia who have blood culture evaluation using Fast Antibiotic Susceptibility Testing vs. standard of care testing: The FAST trial
Trial registration {2a and 2b}.	ClinicalTrials.gov register identifier: NCT06174649
Protocol version {3}	Protocol number 20–0021, version 5.0, 11-APR-2024
Funding {4}	National Institute of Health (NIH), Grant UM1AI104681
Author details {5a}	As above
Name and contact information for the trial sponsor {5b}	Duke University
Role of sponsor {5c}	The study sponsor is Duke University. The research team is responsible for study design, data collection, management, analysis and interpretation of data, writing of the report and dissemination of trial findings.

## Introduction

### Background and rationale {6a}

Gram-negative bacilli (GNB), like *Escherichia coli*, *Klebsiella pneumoniae*, and *Pseudomonas aeruginosa*, cause up to one-third of bloodstream infections (BSIs) worldwide. Ineffective therapy for GNB BSIs is associated with mortality rates in excess of 30% [1–7]. More rapidly determining the antimicrobial susceptibility of bloodstream bacterial isolates from patients with BSI has been proposed as a means to improve clinical outcomes and reduce emergence of bacterial resistance. While novel blood culture diagnostics can provide faster antibiotic susceptibility test results (AST) compared to conventional methods, their clinical impact is unclear and has been assessed in only a few prospective clinical trials [8, 9]. Limitations of prior trials include that they were conducted in areas with low prevalence of antibiotic resistance so most subjects were receiving effective antibiotic therapy at time of enrollment; were not sufficiently powered to detect differences in clinical outcomes; and utilized costly tests with potentially limited applicability to a variety of settings [8, 9].

Unlike these prior studies, the FAST trial will evaluate whether a diagnostic testing strategy involving rapid phenotypic AST testing improves outcomes in patients with GNB BSI in areas with high rates of antibacterial resistance, to assess the clinical impact of rapid AST in settings where it is likely to have the greatest impact.

The rapid AST method that will be used in the FAST trial is VITEK REVEAL™ (bioMérieux Inc., Salt Lake City, USA), which uses small molecule sensor technology to detect growth of bacterial populations by measuring volatile metabolites and provides AST results in ~5 h [10]. VITEK REVEAL™ is approved for clinical use in many regions of the world and provides AST for ~90% of organisms causing Gram-negative BSIs. Use of VITEK REVEAL™ requires little technologist training, and sample preparation takes less than 3 min [10]. The rapid results, scalability, and minimal training requirements of VITEK REVEAL™ make it attractive for use in areas with high GNB resistance.

The VITEK REVEAL™ system has demonstrated good performance. As compared to other methods including Sensititre™ (Thermo Fisher, USA), VITEK^®^2 (bioMérieux, Marcy-l'Étoile, France), and MicroScan™ (Beckman Coulter, USA), the VITEK REVEAL™ system showed 96.3–97.6% categorical agreement (CA) and 96.4–97.7% essential agreement (EA), with 2–3% minor errors (mE), 0.3–1.2% major errors (ME), and 1.2–1.6% very major errors (VMEs) across large collections of antibiotic-susceptible and resistant isolates [11–13].

### Objectives {7}

#### Primary objective

To compare clinical outcomes among subjects with GNB BSI who have bloodstream isolate AST determined using VITEK REVEAL™ vs. SOC methods.

#### Secondary objective

To describe differences in antibiotic utilization up to 3 days post Gram stain result among subjects with GNB BSI who have bloodstream isolate AST determined using VITEK REVEAL™ vs. SOC methods.

#### Exploratory objectives

- To describe discrepancies between VITEK REVEAL™ and SOC methods among subjects randomized to the VITEK REVEAL™ arm
- To describe clinician compliance with AS recommendations in the VITEK REVEAL™ vs. SOC arms
- To describe the desirability of treatment selections in the VITEK REVEAL™ vs. SOC arms using the Desirability of Outcome Ranking for the Management of Antimicrobial Therapy (DOOR-MAT) [14].

### Trial design {8}

This study is a two-arm, multicenter, multinational, prospective, randomized, controlled clinical trial. Hospitalized subjects with GNB in blood cultures will be randomized to have positive blood cultures characterized using SOC AST vs. VITEK REVEAL™ in addition to SOC AST. The primary objective is not to evaluate VITEK REVEAL™ for diagnostic accuracy (which has been approved for clinical use by regulatory agencies in the U.S. and European Union), but rather to determine whether its use in the clinical setting can improve clinical outcomes [11–13].

Eligible subjects will be identified based on results of blood cultures (with growth of GNB) collected for clinical indications. Laboratory testing will occur in local clinical microbiology laboratories. Screening will occur according to eligibility criteria (see below), and eligible subjects will be randomized in a 1:1 ratio to one of two arms. The randomization scheme will be stratified by site and by intensive care unit (ICU) status at the time of the Gram stain result. The primary service, including the treating provider, will be unaware of arm assignment at the time of randomization, so initial antibiotic choice by the treating provider will not be affected by arm assignment. The treating provider for each subject will make all treatment decisions. All blood draws, tests, and procedures will be part of standard of care and no additional tests or procedures will be conducted for study purposes.

After a subject is randomized, information about the subject’s demographics, comorbid conditions, severity of illness, laboratory results, administered antimicrobials, and whether the subject is on comfort care will be obtained from the subject’s medical record (MR) and recorded into the study database. Final outcomes for each enrolled subject will be gathered from the MR.

## Methods: participants, interventions, and outcomes

### Study setting {9}

Hospitalized subjects will be recruited from seven medical centers in India, Israel, Greece, and Spain, all countries with high resistance rates among GNB. A list of study sites can be obtained from Clinicaltrials.gov NCT06174649.

### Eligibility criteria {10}

Hospitalized subjects from participating sites who are screened and meet the inclusion criteria and none of the exclusion criteria will be enrolled in the study. Any eligible subject of any age who has a GNB BSI may be enrolled into this study. Unevaluable subjects will not be replaced and include subjects with erroneous Gram stain results and with SOC cultures that do not yield GNB, death prior to randomization, or history of blood culture with GNB within 7 days prior to randomization. Partial data on unevaluable subjects, including microbiology results and certain baseline data, will be retained.

#### Inclusion criteria

1. Positive blood culture with Gram stain showing GNB
2. Hospitalized at the time of Gram stain result
3. Enrolled within 16 h of blood culture positivity (because VITEK REVEAL™ must be initiated within this time frame per manufacturer’s instructions)

#### Exclusion criteria

1. Positive blood culture for GNB within the prior 7 days (if known at the time of Gram stain result), as prior blood culture result may influence clinical decision making and outcomes
2. Deceased at the time of Gram stain result
3. Gram-positive bacilli, Gram-positive cocci, Gram-negative cocci, yeast, fungi, or multiple morphologies of GNB detected on Gram stain of blood culture
4. Previous enrollment in this study

### Who will take informed consent? {26a}

This study will be conducted with a waiver of informed consent, which is justified because the trial will be evaluating a diagnostic that has received regulatory approval; it will not be evaluating performance and accuracy of the rapid AST method, but rather whether implementation of the rapid AST method as part of routine clinical care impacts patient outcomes. If local and/or national regulations do not allow a waiver of consent, a locally approved consent process will be utilized.

### Additional consent provisions for collection and use of participant data and biological specimens {26b}

The investigators do not expect to collect or use any specimens.

#### Interventions

Initial antibacterial therapy (prior to enrollment or randomization) will be selected at the discretion of the treating clinician at participating sites. Enrolled subjects will be randomized to receive either standard of care testing or VITEK REVEAL™.

For all subjects in both arms, matrix-assisted laser desorption/ionization-time of flight mass spectrometry (MALDI-TOF MS) will be used as the standard clinical organism identification method and local SOC AST of GNB from positive blood cultures will be performed.

All subjects in both arms of the study will undergo standard audit and feedback by institutional Antibiotic Stewardship (AS) programs, which is part of routine clinical care at participating institutions. AS provider notification will be site-specific and occur by telephone, text, or in-person communication. The AS provider will review the subject’s microbiology results and medical records (MRs). The AS provider will follow local or national treatment guidelines and contact the appropriate provider through telephone, text, or in person communication if modifications to therapy are indicated at any time following notification of positive Gram stain result, organism identification, and/or AST results. If the AST result is ready when the AS provider is unavailable (e.g., in the evening or on a weekend), the AS team will review the MRs and make recommendations as soon as possible, but no later than the beginning of the next working day. Escalation and de-escalation recommendations made by AS providers will be recorded in the study database. Compliance with AS recommendations will be recorded. Recommendations can be directly entered into the database by the AS provider or designee to serve as an electronic source when another source document is not available.

In addition to standard audit and feedback, other routine institutional AS activities will continue, such as implementation of institutional treatment guidelines, prior authorization of restricted antibiotics, review of subjects on selected antibiotics with feedback to clinicians, and daily review of specific culture results.

### Explanation for the choice of comparators {6b}

For all subjects in both arms, matrix-assisted laser desorption/ionization time of MALDI-TOF MS will be used as the standard clinical organism identification method and local SOC AST of GNB from positive blood cultures will be performed. SOC AST methods may vary by site and can include VITEK^®^2 (bioMérieux Inc., Salt Lake City, USA), disk diffusion, BD Phoenix™ (BD Diagnostics, USA), or Microscan^®^ (Beckman Coulter, USA). Time from positive culture until organism identification and AST results varies depending on method used and laboratory workflow. Because a prior trial demonstrated that appropriate antibiotic de-escalation was faster when blood culture diagnostics were paired with AS review and feedback^8^, local AS clinicians will review subjects in all arms of the study and recommend antibiotic therapy modifications to treating providers as needed. Prior trials have demonstrated that when SOC methods are used, time to results and time to antibiotic escalation or de-escalation are longer than when newer blood culture diagnostics are used; however, clinical outcomes have not been shown to differ when SOC or rapid blood culture diagnostic tests are used [8, 9].

### Intervention description {11a}

VITEK REVEAL™ is approved for clinical use in the European Union (EU) and in the USA. It provides minimum inhibitory concentrations (MICs) for 23 antibiotics and 10 Gram-negative species [10], that together account for ~90% of organisms causing Gram-negative BSIs.

For subjects randomized to the VITEK REVEAL™ arm, organism ID must be known prior to interpreting AST results using VITEK REVEAL™. VITEK REVEAL™ testing will be performed according to the manufacturer’s instructions and reported in the subject’s medical record and/or reported to the subject’s treating provider. Gram-negative bacilli/antibiotic combinations not reported by VITEK REVEAL™, but routinely reported by the local Microbiology Laboratory using SOC testing, will be added to the final AST report for study subjects. These data will be collected on the eCRF.

Any disagreement in results between the VITEK REVEAL™ and the SOC AST will be reviewed by the Microbiology Laboratory Director and reported in the subject’s eCRF in the study Electronic Data Capture (EDC) system. The primary team caring for the subject will be notified of any disagreement between the VITEK REVEAL™ and the SOC AST results; additional susceptibility testing may be performed per clinical practice, as needed. VITEK REVEAL™ must be initiated within 16 h after the blood culture bottle is flagged positive.

All subjects in both arms of the study will undergo standard audit and feedback by institutional AS programs. The AS provider will review the subject’s microbiology results. The AS provider will follow local or national treatment guidelines and contact the appropriate provider if modifications to therapy are indicated at any time following notification of positive Gram stain result, organism identification, and/or AST results. If the AST result is ready when the AS provider is unavailable (e.g., in the evening or on a weekend), the AS team will review the MRs and make recommendations no later than the beginning of the next working day. Escalation and de-escalation recommendations made by AS providers will be recorded in the study database. Recommendations can be directly entered into the database by the AS provider or designee to serve as an electronic source when another source document is not available.

In addition to standard audit and feedback, other routine institutional AS activities will continue, such as implementation of institutional treatment guidelines, prior authorization of restricted antibiotics, review of subjects on selected antibiotics with feedback to clinicians, and daily review of specific culture results.

### Criteria for discontinuing or modifying allocated interventions {11b}

In sites requiring informed consent, any subject who provided informed consent for participation may withdraw from the study at any time. In sites enrolling with waiver of informed consent there are no pre-specified individual halting rules, although treatment may be modified at any time at the discretion of the treating clinicians. Study withdrawal could also occur if the study or study site is terminated by sponsor for any reason.

### Strategies to improve adherence to interventions {11c}

Recruitment of subjects will occur by site investigators who are clinicians and/or microbiologists who care for persons with bacteremia. Although there are no prespecified strategies that have been developed to increase adherence, subjects will be treated in accordance with local practice at participating sites in order to mirror what might be achievable in real-world practice.

### Relevant concomitant care permitted or prohibited during the trial {11d}

Antibiotic selection is at the discretion of the treating clinicians, who may or may not choose to modify treatment based on the study interventions. Treatment of any co-occurring infections, in addition to bacteremia, is permitted. Subjects may undergo any procedures deemed to be clinically necessary by the treating clinician. Eligibility is limited only by existence of the pre-specified conditions listed in the “Exclusion criteria” section.

### Provisions for post-trial care {30}

Subjects will be followed until death, discharge, or 30 days after randomization, whichever occurs first. No post-trial care will be provided.

### Outcomes {12}

The primary outcome is the composite three-category DOOR outcome up to 30 days after Gram stain result [15, 16].

The primary DOOR outcome measure is defined using three, unweighted ordered levels. From best to worst, they are:

1. Alive without deleterious events
2. Alive with at least 1 deleterious event that is listed in Table 1.
3. Death

**Table 1:** Deleterious events for DOOR outcome

Unsuccessful discharge:	Lack of clinical response:	Undesirable events
• Not discharged from index hospitalization • Readmission to hospital	• Relapse of bacteremia with the same species causing the index infection • Local suppurative complications not present at time of Gram stain result • Seeding distant sites with the same infecting organism after Gram stain result	• Acquisition of hospital-acquired infection – *Clostridioides difficile* - MDRO^1^ • Renal failure that occurs after index bloodstream infection: – Three-fold or greater increase in serum creatinine – New renal replacement therapy

^1^MDRO, multidrug-resistant organism identified on routine clinical or surveillance samples using local laboratory diagnostic procedures and including Methicillin-resistant *Staphylococcus aureus*, Vancomycin-resistant *Enterococcus* species, 3rd generation cephalosporin-non-susceptible Enterobacterales, carbapenem-resistant Enterobacterales, as defined by resistance to imipenem, meropenem, doripenem, or ertapenem OR documentation that the isolate produces a carbapenemase, *Pseudomonas aeruginosa* resistant to carbapenems or multi-drug resistant (resistant to aminoglycosides, cephalosporins, fluoroquinolones, and carbapenems), carbapenem-resistant *Acinetobacter* species, or *Candida auris*

Secondary outcomes include:

- All-cause in-hospital mortality up to 30 days post Gram stain result
- Length of index stay in the hospital up to 30 days post Gram stain result, for those subjects alive at 30 days. Length of stay will be calculated as date of discharge minus date of Gram stain result.
- ICU admission up to 30 days post Gram stain result
- New acquisition of MDRO and/or *Clostridioides difficile* after Gram stain result and up to 30 days post Gram stain result. New acquisition is defined as detection of MDRO/*C. difficile* in subjects who do not have preceding clinical or surveillance cultures with these organisms in the prior 3 months. MDRO will be identified on routine clinical or surveillance samples using local laboratory diagnostic procedures and include:
  ◦ Methicillin-resistant *Staphylococcus aureus*
  ◦ Vancomycin-resistant *Enterococcus* species
  ◦ 3rd generation cephalosporin-non-susceptible Enterobacterales
  ◦ Carbapenem-resistant Enterobacterales, as defined by the CDC: resistant to imipenem, meropenem, doripenem, or ertapenem OR documentation that the isolate produces a carbapenemase
  ◦ *Pseudomonas aeruginosa* resistant to carbapenems/multi-drug resistant (resistant to aminoglycosides, cephalosporins, fluoroquinolones, and carbapenems)
  ◦ Carbapenem-resistant *Acinetobacter* species
  ◦ Candida auris
- Time to effective antibiotic therapy within 3 days from Gram stain result, defined as treatment with an antibiotic (or antibiotic class) to which the blood isolate is susceptible based on SOC AST.
- Time to antibiotic escalation of Gram-negative coverage in those who have antibiotic escalation within 3 days from Gram stain result.
  ◦ Escalation: Changing to a broader spectrum antibiotic (higher number category in Table 2), addition of one or more antibiotics, or conversion of oral (PO) to intravenous (IV) route.
- Time to antibiotic de-escalation of Gram-negative coverage in those who have antibiotic de-escalation within 3 days from Gram stain result.
  ◦ De-escalation: Changing to a narrower spectrum antibiotic (lower number category in Table 2), cessation of one or more antibiotics, or changing from an IV to PO route of appropriate drug (i.e., IV to PO ciprofloxacin or levofloxacin).

**Table 2:** Gram negative antibiotics

**Category**	**Spectrum**	**Antibiotics**
**1**	Narrowest	Ampicillin, cefazolin
**2**	Narrow	Amoxicillin/clavulanic acid, ampicillin/sulbactam, 2nd generation cephalosporins, 3rd generation cephalosporins (except ceftazidime), tetracyclines, trimethoprim/sulfamethoxazole
**3**	Medium	Aminoglycosides (amikacin, gentamicin, tobramycin), aztreonam, ceftaroline, ceftazidime, fluoroquinolones (ciprofloxacin, moxifloxacin, levofloxacin), fosfomycin
**4**	Medium broad	Piperacillin/tazobactam, cefepime, ertapenem
**5**	Broadest	Anti-pseudomonal carbapenems (imipenem/cilastatin, meropenem), ceftazidime/avibactam, ceftolozane/tazobactam, imipenem/relebactam, cefiderocol, tigecycline, meropenem/vaborbactam, colistin, polymyxin B

Exploratory outcomes include:

- The five-category DOOR up to 30 days after Gram stain result. The five-ordered levels of DOOR clinical outcomes from best to worst are:
  ◦ Alive without deleterious events (listed in Table 1)
  ◦ Alive with 1 deleterious event
  ◦ Alive with 2 deleterious events
  ◦ Alive with 3 deleterious events
  ◦ Death
- Categorical agreement between VITEK REVEAL™ and SOC testing (among subjects randomized to VITEK REVEAL™ arm only).
- Clinician compliance with AS recommendations measured by changes in antibiotics administered in accordance with at least one of the AS escalation/de-escalation recommendations.
- Desirability of treatment selections based on antibiotic spectrum and activity in relation to bloodstream isolate susceptibility profiles (DOOR MAT)^13^.

### Participant timeline {13}

Subjects will be followed until death, discharge, or 30 days after randomization, whichever occurs first. There will be no study-specific visits. The schedule of events is shown in Table 3.

**Table 3:** Schedule of activities

**Activity**	**Screening**	**Enrollment/baseline (Day 0)**	**Day 1**	**Day 2**	**Day 3**	**Day 30**
Record date & time of gram stain result	x					
Evaluation of inclusion/exclusion criteria	x					
Randomization		x				
Perform AST based on assigned arm^1^		x				
Record demographics		x^2^				
Record medical history		x^2^				
Record components of qPitt		x^2^				
Record comfort care		x^2^				
Record antibiotic administration or other medication details		x^2^	x^2^	x^2^	x^2^	
Record admission information		x^3^				
Record source of bacteremia		x^2^	x^2^	x^2^	x^2^	x^4^
Record results of blood culture organism identification			x^2^	x^2^	x^2^	x^4^
Record results of AST			x^2^	x^2^	x^2^	x^4^
Record AS provider escalation/de-escalation recommendation			x^2^	x^2^	x^2^	
Record date of discharge						x
Record DOOR events/outcomes						x
Record readmission occurrence						x

^1^VITEK REVEAL™ AST must be initiated within 16 h after the blood culture bottle is flagged positive

^2^Data can be recorded for a subset of day 0 data and day 1, 2, and 3 time points at the final visit by reviewing the MR

^3^ICU status must be collected and recorded at day 0 for randomization

^4^Including results from day 4 up to day 30

### Sample size {14}

Clinical outcomes up to 30 days post-Gram stain result will be compared using the DOOR. Data from the MERINO [17] and RAPIDS-GN [9] trials were used to inform the expected DOOR distribution.^,^ A nonparametric Wilcoxon rank sum test based on the DOOR distribution will be employed to test the null hypothesis that outcomes in the VITEK REVEAL™ arm are not better than in the SOC arm, where this is defined as DOOR probability < 50%, where DOOR probability is calculated by Pr (more desirable outcome with rapid testing than SOC) + 1/2Pr (outcome with rapid testing is equal to SOC) compared to the alternative hypothesis that outcomes in the VITEK REVEAL™ arm are better than in the SOC arm. Based on the data from the RAPIDS-GN and MERINO trials, in the SOC arm, the following is expected: a 10–12% mortality rate, about 32% of subjects alive with deleterious events, and the remaining 56–58% of subjects alive without events. Assuming the difference between the arms in mortality is 4–5% and in being alive with no events is 9%, the DOOR probability is calculated to be between 54.8 and 55.1%. With 900 enrolled subjects (450 in each arm) and an unevaluable rate of 10%, we will have at least 405 evaluable subjects in each arm. This will provide at least 80% power to reject the null hypothesis with 2.5% significance level for a one-sided test. Assuming that approximately 11% of bloodstream isolates will be “off-panel” and the rapid testing failure rate will be 11%, the planned sample size provides at least 70% power to detect a difference in the hypothesized DOOR distribution between the SOC and VITEK REVEAL™ arm.

Since the trial is intended to evaluate utility of the rapid testing strategy in areas with high prevalence of antibiotic- resistant infections, and resistance is expected to vary by participating site, we will evaluate antibiotic resistance of blood isolates from enrolled subjects as the trial progresses. Enrollment may be capped at sites that are predominantly enrolling subjects with antibiotic-susceptible infections, to ensure accrual of cephalosporin and carbapenem-resistant and susceptible isolates.

### Recruitment {15}

We plan to conduct this trial over 18 months. The anticipated enrollment period is December 2023–June 2025. At the time of manuscript submission, this trial is currently enrolling. Participants will be recruited from seven sites in Greece, India, Israel, and Spain.

## Assignment of interventions: allocation

### Sequence generation {16a}

Computer-generated, permuted block randomization with block size of 4 will be used for allocation. The randomization scheme will be stratified by site for all sites and by intensive care unit (ICU) status at the time of the Gram stain result. These stratification factors were selected to balance arms for site-specific factors (e.g., SOC method, clinical management, and stewardship recommendations) and critical illness, which may impact outcomes.

### Concealment mechanism {16b}

Participants will be randomized using RAVE, a web-based Medidata platform. Allocation concealment will be ensured, as the randomization list will be generated and kept centrally by a study statistician. The list will be kept confidential and allocation communicated to sites electronically via an online enrollment module once a subject has been enrolled.

### Implementation {16c}

Subjects meeting enrollment criteria will be assigned, in a 1:1 ratio to one of the two arms: (1) SOC AST Control Arm or (2) VITEK REVEAL™ Arm. The randomization process will be managed via an online enrollment module within RAVE, the electronic data management system. Laboratory technologists at each site will screen subjects for eligibility and randomize those meeting inclusion criteria and no exclusion criteria using the electronic data management system. Screening and randomization will occur as soon as possible after a blood culture with GNB on Gram staining is noted in the laboratory.

## Assignment of interventions: blinding

### Who will be blinded {17a}

The primary service, including the treating provider, will be unaware of randomization assignment at the time of randomization; initial antibiotic choice will not be affected by arm assignment. Once VITEK REVEAL™ results become available and/or AS recommendations are made, treating providers may become aware of randomization assignment. The AS team and laboratory technologists will not be blinded. The statistical team and outcomes assessors will not be blinded to arm assignment.

### Procedure for unblinding if needed {17b}

Not applicable as site investigators and treating clinicians will be aware of study arm.

## Data collection and management

### Plans for assessment and collection of outcomes {18a}

Demographic, clinical, and laboratory data will be collected from the medical record and recorded via electronic case report form (eCRF). The site staff who will be entering data into the EDC will receive training on the system, after which each person will be issued a unique user identification and password. Site personnel who have not undergone training may not use the system and will not be issued user identification and password until appropriate training is completed.

Quality assurance reports will be generated to ensure study data are clean, accurate, and complete. Quality assurance reports will include, but are not limited to, the following: missing forms, missing and out-of-range values, automated data queries, and targeted manual reviews of study data.

### Plans to promote participant retention and complete follow-up {18b}

Subjects recruited from sites requiring informed consent may withdraw their consent for study participation at any time without penalty. An investigator may also withdraw a subject from participating in the study for any reason. Subjects who withdraw, are withdrawn, or are lost to follow-up after administration of the study interventions will not be replaced. Subjects will be considered clinically evaluable if they meet all inclusion criteria and no exclusion criteria, have a primary outcome assessment, and do not have any missing data or major protocol violations which prevent evaluation of their outcome.

### Data management {19}

eCRFs specific to this study have been developed for use by site study staff. The sponsor’s monitoring staff will either conduct site visits or remote source verification to assure veracity and completeness of data. Any discrepancies in data collection will trigger site retraining for the relevant data fields.

### Confidentiality {27}

Personal health information will be collected and stored securely within the electronic study database for a minimum of 6 years after study completion.

### Plans for collection, laboratory evaluation, and storage of biological specimens for genetic or molecular analysis in this trial/future use {33}

Blood culture collection and SOC testing will be conducted in accordance with local clinical procedures. VITEK REVEAL™ testing will be done in accordance with manufacturer’s guidelines. No samples will be stored or collected.

## Statistical methods

### Statistical methods for primary and secondary outcomes {20a}

Analyses will be performed based on the intention-to-treat principle. The number of events and number and percentage of subjects with events in each arm will be tabulated and presented. For continuous variables, summary statistics will include number of subjects, number of subjects with missing data, mean and standard deviation, median (Q1-Q3), and minimum and maximum. For categorical variables, summary statistics will include number and percentage for each category. We will use the Wilcoxon rank sum test for comparing continuous outcomes and Fisher’s exact test or Pearson’s chi-squared test for comparing categorical outcomes. The difference in the proportion of the number and percentage of subjects with events between the arms will be calculated with the corresponding 95% confidence interval.

The DOOR will be summarized using the DOOR probability [15]. The DOOR probability is estimated by Wilcoxon-Mann-Whitney statistics corrected for ties, divided by the product of the number of volunteers in each group, along with a 95% confidence interval [18]. Individual components included in DOOR will be analyzed and examined separately.

### Interim analyses {21b}

There is no planned interim efficacy analysis involving a statistical test of the primary endpoint. As part of the benefit-risk analysis, one interim analysis is planned when at least 50% of subjects have been enrolled and up to 30 days of follow-up have elapsed. The predictive interval plots (PIPS) method will be used at an interim time point to predict 95% confidence intervals of the treatment effect at the end of the study [19, 20]. PIPS will be created for the primary endpoint based on the data collected up to the interim time point. Results of the interim analysis will not be shared with the site investigators prior to the completion of the trial. The DSMB will advise the sponsor on whether to continue, modify, or terminate the trial based on risk assessment during the interim analysis [21, 22].

### Methods for additional analyses (e.g., subgroup analyses) {20b}

Treatment interactions with severity of illness based on the source of bacteremia and the qPitt will be evaluated to inform subgroup analyses. Subgroup analyses will be performed to evaluate the rapid testing method among subjects (1) across geographic locations by site and country, (2) whose isolates have different phenotypic resistance patterns, (3) with infections due to on-panel Gram-negative species (species included in the VITEK REVEAL™ panel), and (4) with different clinical and demographic characteristics including in ICU at randomization, immunocompromised status, low vs. high Charlson scores, sex, and age.

### Methods in analysis to handle protocol non-adherence and any statistical methods to handle missing data {20c}

Sites will be required to document all protocol violations within the electronic case report form, including the nature of the deviation, actions taken to resolve or avoid recurrence of the deviation, and date major deviations were reported to the ethics committee, if indicated.

Incomplete and inconsistent results identified during monitoring review will be queried. All efforts will be made to minimize the missing data in the study database. Number of missing data for each domain will be summarized. The effect that any missing data might have on results will be assessed via sensitivity analysis. If the pattern of missing data is different from the assumption, further sensitivity analyses will be provided that are tailored to the missing data pattern observed. Given the nature of data collection, minimal missingness is expected. The primary analyses of DOOR outcomes will use available DOOR values in the ITT population. Sensitivity analyses of DOOR will include methods to account for missing data. All other analyses will be based on available results.

### Plans to give access to the full protocol, participant-level data, and statistical code {31c}

The datasets analyzed during the current study and statistical code are available from the corresponding author on reasonable request, as is the full protocol.

## Oversight and monitoring

### Composition of the coordinating center and trial steering committee {5d}

Duke University serves as the overall study sponsor, responsible for trial conduct and safety oversight, site training, monitoring, and close oversight of study visits to assure proper adherence to trial protocol and research standards.

### Composition of the data monitoring committee, its role and reporting structure {21a}

The FAST trial will be reviewed at least annually by a Data Safety Monitoring Board (DSMB). The DSMB membership will be composed of investigators and statisticians that are not currently associated with the FAST protocol. The DSMB will review accumulating data from FAST by treatment arm to ensure validity and integrity of the trial and make a benefit-risk assessment. Safety events will not be followed or recorded as deleterious events from the VITEK REVEAL™ platform are not expected. The DSMB will review study progress and focus on data quality, enrollment, and implementation of the SOC and VITEK REVEAL™ strategies. The study statisticians will prepare a report for the DSMB that will include baseline summaries, data quality and completeness, and will address issues concerning viability and appropriate execution of the protocol such as accrual and invalid or unobtainable results via either SOC or VITEK REVEAL™ by site and by organism. The DSMB members will be separate and independent of research personnel participating in this study and will not have scientific, financial, or other conflicts of interest related to this trial. The DSMB will review data at prespecified intervals during the trial and will conduct ad hoc reviews, as appropriate for immediate concerns regarding observations during the trial.

### Adverse event reporting and harms {22}

Safety events will not be followed or recorded as deleterious events from VITEK REVEAL™ are not expected.

### Frequency and plans for auditing trial conduct {23}

Monitoring for this study will be overseen by a Contract Research Organization, which will conduct periodic site visits throughout the trial, including a review of data collection forms, source data verification, adverse event reporting, and consent documentation. The monitors will also count and view the study product to verify the number of reagents.

### Plans for communicating important protocol amendments to relevant parties (e.g., trial participants, ethical committees) {25}

Protocol modifications will be submitted for review by participating institutional review boards (IRBs) for approval prior to implementation.

### Dissemination plans {31a}

Study results will be reported in accordance with Consolidated Standards of Reporting Trials guidelines for randomized controlled trials. Results will be submitted to clinicaltrials.gov within 1 year of study completion. The authors plan to submit study results for publication in a peer-reviewed scientific journal and/or present at relevant conferences. No participant-protected health information will be revealed in any publication or presentation.

## Discussion

Bloodstream infections caused by GNB cause significant morbidity and mortality worldwide, particularly in areas with high antibacterial resistance. Rapid AST methods for bloodstream infections have potential to improve patient outcomes by facilitating prompt, targeted antibiotic therapy. However, while novel and rapid blood culture diagnostics can provide faster organism AST compared to standard methods, their clinical impact is unclear and has been assessed in only a few prospective clinical trials [8, 9]. The recently completed RAPIDS-GN trial was a randomized controlled trial (RCT) conducted at two United States (US) centers to compare clinical impact of a rapid identification (ID) and phenotypic AST method vs. standard of care (SOC) methods in subjects with GNB BSI [9]. RAPIDS-GN demonstrated that compared to conventional testing, a rapid phenotypic AST method implemented with antimicrobial stewardship (AS) clinician review led to significantly faster antibiotic modifications for treatment of GNB-BSI, and enabled antibiotic escalations to occur almost 2 days faster for subjects with antibiotic-resistant infections. The RAPIDS-GN trial found that differences in testing strategies did not impact clinical outcomes but was conducted in areas with low prevalence of antibiotic resistance and was powered to detect differences in antibiotic utilization rather than clinical outcomes.

The FAST trial will address these limitations and evaluate whether use of a rapid phenotypic AST method improves outcomes compared to use of conventional methods *in clinical settings with high antibacterial resistance.* Results of this study have implications for diagnosis, management, and outcomes of GNB and will inform future efforts to develop blood culture diagnostics and integrate them into clinical care. Findings will be applicable to regions of the world with high endemic drug resistance among GNB.

While phenotypic and genotypic methods for antibiotic resistance detection are commercially available, phenotypic AST methods are optimal for capturing the multiple, diverse resistance mechanisms present among GNB. It is likely impossible to design a genotypic platform capable of detecting all the resistance genes that may be present among GNB. The rapid phenotypic AST method that will be used in the FAST trial is VITEK REVEAL™ (bioMérieux Inc., Salt Lake City, USA), which uses small molecule sensor technology to detect growth of bacterial populations by measuring volatile metabolites and provides AST results in ~5 h. VITEK REVEAL™ is approved for clinical use in many regions of the world and provides AST for ~90% of organisms causing Gram-negative BSIs. Use of VITEK REVEAL™ requires little technologist training, and 3 min of sample preparation time, and it is scalable, making it attractive for many settings, including busy clinical laboratories and low-resource settings [10].

The VITEK REVEAL™ system has demonstrated good performance. As compared to other methods including Sensititre™ (Thermo Fisher, USA), VITEK^®^2 (bioMérieux, Marcy-l'Étoile, France), and MicroScan™ (Beckman Coulter, USA), the VITEK REVEAL™ system showed 96.3–97.6% categorical agreement (CA) and 96.4–97.7% essential agreement (EA), with 2–3% minor errors (mE), 0.3–1.2% major errors (ME), and 1.2–1.6% very major errors (VMEs) across large collections of antibiotic-susceptible and resistant isolates [11–13].

## Trial status

The trial enrolled its first subject on December 27, 2023, and recruitment was ongoing at the time of manuscript submission. The most current protocol version number is 20–0021, version 5.0 dated 11 April 2024.

## Data Availability

Upon completion of the study, final de-identified data may be supplied on request, following submission of a plan for data use, approval of the plan by the Antibacterial Resistance Leadership Group (ARLG), and execution of required institutional agreements.

## Abbreviations

ARLG: Antibacterial Resistance Leadership Group
AS: Antimicrobial stewardship
AST: Antimicrobial susceptibility testing
BSI: Bloodstream infection
CA: Categorical agreement
CDC: Centers for Disease Control and Prevention
CRO: Contract Research Organization
DMID: Division of Microbiology and Infectious Diseases
DOOR: Desirability of Outcome Ranking
DOOR MAT: Desirability of Outcome Ranking for the Management of Antimicrobial Therapy
DSMB: Data and Safety Monitoring Board
EA: Essential agreement
eCRF: Electronic Case Report Form
EDC: Electronic Data Capture
EU: European Union
EUCAST: European Committee on Antimicrobial Susceptibility Testing
FAST: Fast Antibiotic Susceptibility Testing for Gram-Negative Bacteremia Trial
GNB: Gram-negative bacilli
ICU: Intensive care unit
ID: Identification
IRB: Institutional review board
ITT: Intention-to-Treat
IV: Intravenous
MALDI-TOF MS: Matrix-assisted laser desorption/ionization time of flight mass Spectrometry
MDRO: Multi-drug-resistant organism
ME: Major error
mE: Minor error
MIC: Minimum inhibitory concentration
MOP: Manual of Procedures
MR: Medical Record
NIAID: National Institute of Allergy and Infectious Diseases
NIH: National Institutes of Health
PIPS: Predictive interval plots
PO: Oral
qPitt: Quick Pitt Bacteremia Score
RCT: Randomized controlled trials
SD: Standard deviation
SDMC: Statistics and Data Management Center
SOC: Standard of Care
US: United States
VME: Very major error
